# Methylglyoxal Detoxification Revisited: Role of Glutathione Transferase in Model Cyanobacterium *Synechocystis* sp. Strain PCC 6803

**DOI:** 10.1128/mBio.00882-20

**Published:** 2020-08-04

**Authors:** Xavier Kammerscheit, Arnaud Hecker, Nicolas Rouhier, Franck Chauvat, Corinne Cassier-Chauvat

**Affiliations:** a Université Paris-Saclay, CEA, CNRS, Institute for Integrative Biology of the Cell (I2BC), Gif-sur-Yvette, France; b Université de Lorraine, INRAE, IAM, Nancy, France; University of Delaware

**Keywords:** cyanobacteria, detoxification, enzyme assay, glutathione transferase, glyoxalase pathway, *in vivo* analysis, methylglyoxal, oxidative stress, sugar metabolism

## Abstract

Methylglyoxal (MG) is a detrimental metabolic by-product that threatens most organisms (in humans MG causes diabetes). MG is predominantly detoxified by the glyoxalase pathway. This process begins with the conjugation of MG with glutathione (GSH), yielding a hemithioacetal product that is subsequently transformed by the glyoxalase enzymes into d-lactate and GSH. MG has been overlooked in photosynthetic organisms, although they inevitably produce it not only by the catabolism of sugars, lipids, and amino acids, as do heterotrophic organisms, but also by their active photoautotrophic metabolism. This is especially true for cyanobacteria that are regarded as having developed photosynthesis and GSH-dependent enzymes to detoxify the reactive oxygen species produced by their photosynthesis (CO_2_ assimilation) and respiration (glucose catabolism), which they perform in the same cell compartment. In this study, we used a combination of *in vivo* and *in vitro* approaches to characterize a logical, but as yet never described, link between MG detoxification and a (prokaryotic) representative of the evolutionarily conserved glutathione transferase (GST) detoxification enzymes. We show that the Sll0067 GST of the model cyanobacterium *Synechocystis* sp. strain PCC 6803 plays a prominent role in MG tolerance and detoxification, unlike the other five GSTs of this organism. Sll0067 catalyzes the conjugation of MG with GSH to initiate its elimination driven by glyoxalases. These results are novel because the conjugation of MG with GSH is always described as nonenzymatic. They will certainly stimulate the analysis of Sll0067 orthologs from other organisms with possible impacts on human health (development of biomarkers or drugs) and/or agriculture.

## Abstract (precis)

In most organisms, methylglyoxal (MG), a toxic metabolite by-product that causes diabetes in humans, is predominantly detoxified by the glyoxalase enzymes. This process begins with the so-called “spontaneous” conjugation of MG with the cytoprotectant metabolite glutathione (GSH). In this study, we unravel a logical, but as yet unsuspected, link between MG detoxification and a (prokaryotic) representative of the ubiquitous glutathione transferase (GST) enzymes. We show that a GST of a model cyanobacterium plays a prominent role in the detoxification of MG in catalyzing its conjugation with GSH. This finding is important because this reaction, always regarded as nonenzymatic, could exist in plants and/or human and thus have an impact on agriculture and/or human health.

## INTRODUCTION

Methylglyoxal (MG) is a very dangerous dicarbonyl compound that strongly interacts with lipids, nucleic acids, and the lysine and arginine residues of proteins, generating advanced glycation end products (AGEs) that strongly disturb cell metabolism in prokaryotes (1) and eukaryotes (2, 3). In fact, MG has a dual nature depending on its concentrations within the cells, acting in signaling at low concentrations while provoking detrimental effects at high concentrations (2, 4). In humans, MG is implicated in diabetes and age-related disorders, such as retinopathy, nephropathy, cancer, and Parkinson’s and Alzheimer’s diseases (3), and MG is increasingly regarded as a marker of diabetes-related diseases. In plants, MG is thought to play signaling roles via Ca^2+^, reactive oxygen species (ROS), K^+^, and abscisic acid, and these processes are thought to provide the foundation for developing stress-resilient crops capable of coping with rapidly changing environments (2).

**FIG 1:**
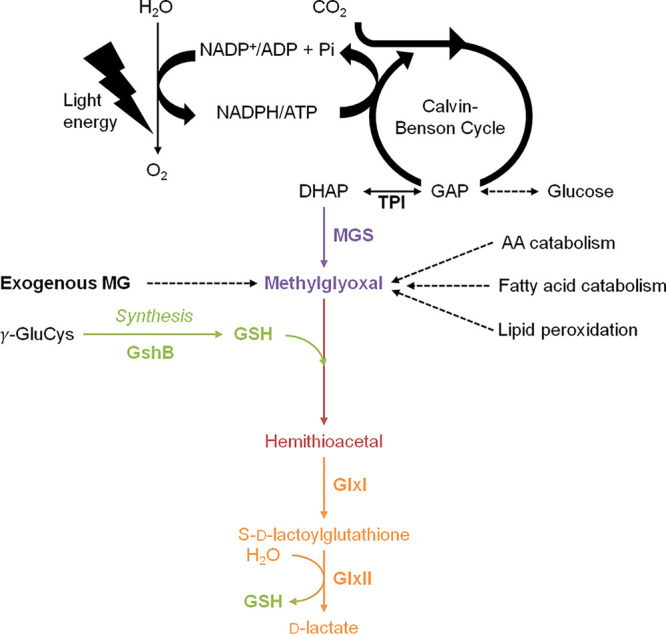
Schematic representation of the production and glutathione-dependent detoxification of methylglyoxal. In photosynthetic organisms such as cyanobacteria, methylglyoxal (MG) is inevitably produced from the light-driven assimilation of CO_2_ and the catabolism of glucose that generates the triose phosphate isomers (TPI) glyceraldehyde-3-phosphate (GAP) and dihydroxyacetone phosphate (DHAP), which can produce MG spontaneously or through the MG synthase (MGS) enzyme. MG is also produced by lipid peroxidation and the catabolism of amino acids (AA) and fatty acids. MG is mainly detoxified by the GSH-dependent glyoxalase pathway. The first step, always presented as spontaneous, involves the conjugation of glutathione (GSH) with MG, forming hemithioacetal (HTA), which is subsequently isomerized to *S*-d-lactoylglutathione by glyoxalase I (GlxI) and hydrolyzed by glyoxalase II (GlxII) to release GSH and d-lactate.

MG is mainly formed by the nonenzymatic breakdown of the triose phosphate isomers dihydroxyacetone phosphate (DHAP) and glyceraldehyde-3-phosphate (G3P) (1, 3), which rapidly lose α-carbonyl protons and their phosphate groups, generating MG (Fig. 1). MG is also generated by the spontaneous auto-oxidation of ketone bodies and sugars, the Maillard reaction between reducing sugars and amino acids, and lipid peroxidation. In addition, various enzymes generate MG from (i) the aminoacetone produced by glycine and threonine metabolisms (monoamine oxidase), (ii) the fatty acid-derived acetone (cytochrome P450), and (iii) the elimination of an inorganic phosphate from DHAP (MG synthase [MGS]) (2, 3). MG can be detected directly through derivatization reactions performed under acidic conditions to avoid the spontaneous production of MG from DHAP and G3P (2, 4).

MG is predominantly detoxified by the glyoxalase pathway (Fig. 1), which starts by the so-called “spontaneous” (nonenzymatic) conjugation of MG with glutathione (GSH) to form a hemithioacetal (HTA). Then, HTA is isomerized by glyoxalase I (GlxI; *S*-d-lactoylglutathione lyase; EC 4.4.1.5) to *S*-d-lactoylglutathione (S-lactoylGSH) that is hydrolyzed by glyoxalase II (GlxII; *S*-2-hydroxyacylglutathione hydrolase; EC 3.1.2.6) to release d-lactate and GSH. MG can also be detoxified by the following enzymes: MG dehydrogenase, aldehyde dehydrogenases, aldo-keto reductases, α-dicarbonyl/l-xylulose reductase, and the MG reductase (3, 4).

Little attention has been paid so far to MG metabolism in photosynthetic organisms, even though they inevitably produce MG by their active photosynthetic assimilation of CO_2_ (2, 5, 6), explaining why plant genomes possess multiple *glxI* and *glxII* genes (2). In contrast, heterotrophic organisms from Escherichia coli to humans possess only one copy of each *glxI* or *glxII* gene (7). MG production, signaling, and detoxification systems are of primary importance in cyanobacteria, the environmentally important prokaryotes (8), because they perform the two MG-producing pathways, photosynthesis (CO_2_ fixation and gluconeogenesis) and respiration (glucose catabolism), in the same cell compartment (6). Furthermore, cyanobacteria are regarded as the inventor of oxygenic photosynthesis (9, 10), and GSH and GSH-utilizing enzymes such as glutathione transferases (GSTs), to cope with the ROS often produced by their active photosynthesis (11). Attesting to the importance of GST in cyanobacteria, the well-studied unicellular model *Synechocystis* sp. strain PCC 6803 endowed with a small genome (about 4 Mb), possesses six GST (Sll0067, Sll1147, Sll1545, Sll1902, Slr0236, and Slr0605). We previously showed that Sll1545 and Slr0236 operate in the protection against photo-oxidative stress triggered by high light or H_2_O_2_ (12) and that Sll1147 and its human orthologs play a prominent role in the tolerance to membrane stresses triggered by heat, cold, and lipid peroxidation (13). Concerning Sll0067, we report here that it operates in the protection against MG, unlike the other five GSTs. Consistently, we show that Sll0067 catalyzes the conjugation of GSH with MG, the first step in MG detoxification by the Glx enzymes. These findings are important because the conjugation of GSH with MG is always described as spontaneous (nonenzymatic) in all organisms (2–4). Our report will likely stimulate research on MG signaling and detoxification in humans and animals (with a possible influence on the identification of biomarkers and drugs), plants (with possible influence on agriculture), and cyanobacteria (with influence on the production of carbon-based chemicals, such as lactate) (14).

## RESULTS

### Sll0067 is dispensable to the photoautotrophic growth of *Synechocystis* sp. strain PCC 6803, but it operates in the protection against methylglyoxal.

**FIG 2:**
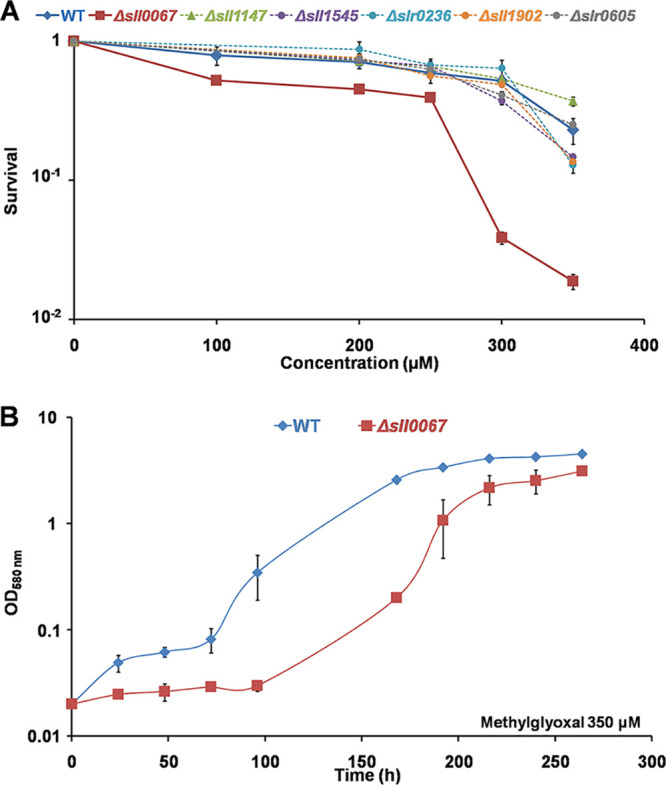
The Δ*sll0067* mutant is sensitive to methylglyoxal, unlike the other five GST deletion mutants. (A) Typical survivals of the WT and all six GST deletion mutants (Δ*sll0067*, Δ*sll1147*, Δ*sll1545*, Δ*sll1902*, Δ*slr0236*, and Δ*slr0605*) exposed for 72 h to various MG concentrations. (B) Typical growth curves of the WT and Δ*sll0067* mutant strains incubated in liquid MM containing 350 μM MG. The data shown in both panels represent the means ± standard deviation (SDs) of three experiments.

To analyze the role of Sll0067 in *Synechocystis* sp. strain PCC 6803, we constructed a Δ*sll0067*::Km^r^ deletion mutant and verified by PCR (see Tables S1 and S2 and Fig. S1 in the supplemental material) that the kanamycin resistance gene (Km^r^) marker had properly replaced *sll0067* in all 10 copies of the polyploid chromosome (15). All Δ*sll0067*::Km^r^ transformants grew as healthy as the wild-type (WT) strain (Fig. 2) and possessed only Δ*sll0067*::Km^r^ chromosomes (Fig. S1). The absence of WT chromosomes in the Δ*sll0067*::Km^r^ mutant (here called Δ*sll0067*) was confirmed by studying cells grown for multiple generations in the absence of Km (absence of counterselection of WT, i.e., Km^s^ chromosome copies). Collectively, these data demonstrate that *sll0067* is not essential for the standard photoautotrophic growth of *Synechocystis* sp. strain PCC 6803.

10.1128/mBio.00882-20.1 TABLE S1 Characteristics of genes and plasmids used in this study. The conditional expression vector for the cyanobacteria *Synechocystis* sp. strains PCC6803 and PCC6714 or *Synechococcus* sp. strains PCC7942 and PCC6301 was described previously (30). Download Table S1, DOCX file, 0.01 MB. Copyright © 2020 Kammerscheit et al. 2020 Kammerscheit et al. This content is distributed under the terms of the Creative Commons Attribution 4.0 International license.

10.1128/mBio.00882-20.2 TABLE S2 List and characteristics of the PCR primers used in this study. FW. forward; RV. reverse. Numbers in brackets correspond to those used in Fig. S1 and S2 in the supplemental material. Download Table S2, DOCX file, 0.01 MB. Copyright © 2020 Kammerscheit et al. 2020 Kammerscheit et al. This content is distributed under the terms of the Creative Commons Attribution 4.0 International license.

10.1128/mBio.00882-20.4 FIG S1 The *sll0067* gene is dispensable to the photoautotrophic growth of *Synechocystis* sp. strain PCC 6803. (A) Schematic representation of the s*ll006*7 chromosome locus in the WT strain and the Δ*sll0067*::Km^r^ mutant constructed in this study. The *sll0067* and Km^r^ genes are represented by blue and red arrows, respectively. The same colors represent the corresponding PCR primers (dotted arrows, Table S2) and PCR products (double arrows) typical of the presence of WT (*sll0067^+^*) or mutant (Δ*sll0067*::Km^r^) chromosome copies. (B) Typical UV-light image of the agarose gel showing the PCR products corresponding to the genes *sll0067^+^* (PCR2, blue [see the 187-bp band]) or Km^r^ (PCR1, red [see the 941-bp band]) generated from the WT strain and the Δ*sll0067*::Km^r^ mutant grown in absence (MM for mineral medium) or presence of Km. M, marker DNA (GeneRuler 1-kb Plus DNA ladder; Thermo Scientific). (C) Typical growth curve (initial OD_580_ = 0.02) of WT and Δ*sll0067*::Km^r^ strains incubated under standard conditions. Download FIG S1, TIF file, 0.4 MB. Copyright © 2020 Kammerscheit et al. 2020 Kammerscheit et al. This content is distributed under the terms of the Creative Commons Attribution 4.0 International license.

Next, the influence of various stresses on the growth and survival of the Δ*sll0067* mutant and the WT strain were tested. The Δ*sll0067* mutant was not affected by either photo-oxidative stresses (high light, H_2_O_2_, menadione, or methylene blue), unlike the Δ*sll1545*, Δ*slr0236*, and Δ*sll1545*-Δ*slr0236* mutants (12), or temperature stresses (heat or cold) or *n*-*tert*-butyl hydroperoxide, unlike the Δ*sll1147* mutant (13).

Very interestingly, the Δ*sll0067* mutant appeared to be sensitive to exogenous MG (Fig. 2), unlike the other five GST-lacking mutants (Δ*sll1147*, Δ*sll1545*, Δ*sll1902*, Δ*slr0236*, and Δ*slr0605*), the construction and analysis of which have been already reported (12, 13) or will be published elsewhere (in the case of the Δ*sll1902* and Δ*slr0605* mutants). Collectively, these findings indicate that Sll0067 is specifically involved in the protection against MG.

### The MG-sensitive Δ*sll0067* mutant exposed to exogenous MG (or glucose) accumulates MG.

The role of Sll0067 in MG resistance was studied by measuring the intracellular content of MG in the Δ*sll0067* and WT strains incubated with or without MG, using a standard assay based on the derivatization of MG with the 5,6-diamino-2,4-dihydroxypyrimidine sulfate dihydrate (DDP) chemical that generates the 2-methylumazine fluorescent product (16, 17). To validate this assay in *Synechocystis* sp. strain PCC 6803, we verified that it could measure the difference in the intracellular MG content of relevant strains altered in MG production or elimination. These control strains were the GSH-depleted mutant (Δ*gshB*) previously reported (18) and the MG synthase deletion mutant (Δ*mgs*) presently constructed (Fig. S2); both strains grow well under standard conditions (18; see also Fig. S2).

10.1128/mBio.00882-20.5 FIG S2 The *mgs* gene is dispensable for the photoautotrophic growth of *Synechocystis* sp. strain PCC 6803. (A) Schematic representation of the *sll0036* (*mgs*) chromosome locus in the WT strain and the Δ*mgs*::Km^r^ mutant constructed in this study. The genes and their corresponding PCR primers (Table S2) and PCR products are represented by colored arrows (*mgs*, blue; Km^r^, red). (B) Typical UV-light image of the agarose gel showing the PCR products corresponding to the *mgs* (PCR1, blue) and Km^r^ (PCR2, red) generated from the WT strain and the mutant Δ*mgs*::Km^r^ grown without or with Km. M, the 1-kb DNA extension ladder (Invitrogen). (C) Typical growth curve (initial OD_580_ = 0.02) of WT and Δ*mgs*::Km^r^ strains incubated under standard conditions. All experiments were performed at least three times (error bars indicate the standard deviations). Download FIG S2, TIF file, 0.3 MB. Copyright © 2020 Kammerscheit et al. 2020 Kammerscheit et al. This content is distributed under the terms of the Creative Commons Attribution 4.0 International license.

**FIG 3:**
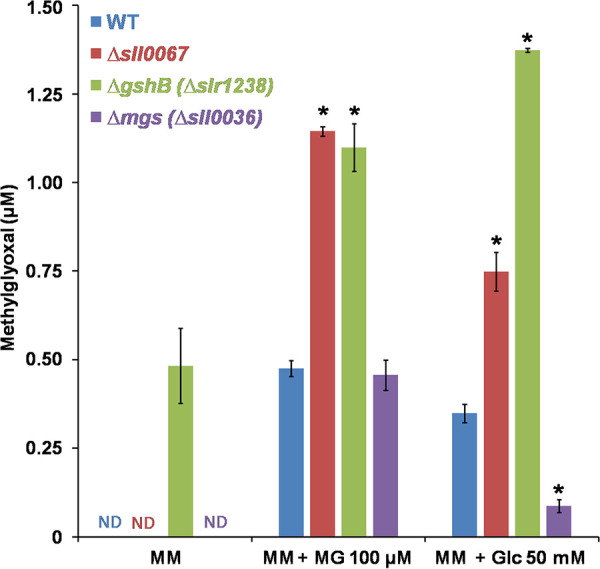
Influence of exogenous MG, or glucose, on the intracellular MG level in various strains of *Synechocystis* PCC 6803. Portions (100 ml) of mid-log-phase cultures, incubated for 18 h in standard liquid MM without or with 100 μM methylglyoxal (MG) or 50 mM glucose (Glc), were used to measure the intracellular concentration of MG in the WT strain or the mutants lacking Sll0067 (Δ*sll0067*), MG synthase (Δ*mgs*), or depleted in GSH (Δ*gshB*). The data are expressed as means ± the SD of three experiments. ND, not detected; *, significant difference between mutants and the WT (*t* test, *P* < 0.05).

In the absence of exogenous MG, no MG was observed in the WT, Δ*mgs*, and Δ*sll0067* strains (Fig. 3), whereas MG was abundant in the Δ*gshB* mutant (Fig. 3) lacking GSH that is required for MG removal (3, 4, 6). In response to exogenous MG all studied strains WT, Δ*mgs*, Δ*gshB*, and Δ*sll0067* accumulated MG (Fig. 3). The levels were similar in WT and Δ*mgs* strains, in agreement with MG synthase playing no role in MG uptake or elimination. MG accumulation was higher in the Δ*gshB* and Δ*sll0067* mutants that are hypersensitive to MG (18; see also Fig. 2). These data indicate that, similar to GSH, Sll0067 is required for MG removal (Fig. 3).

MG accumulation was also observed in all WT, Δ*mgs*, Δ*gshB*, and Δ*sll0067* strains incubated with glucose, which stimulates glucose catabolism (19), a process generating MG. As anticipated, the MG levels were low in the Δ*mgs* mutant (Fig. 3) and higher in the Δ*gshB* and Δ*sll0067* mutants, confirming that Sll0067 operates in MG removal, like GSH (Fig. 3).

### The Δ*sll0067* mutant exposed to exogenous MG accumulates GSH in addition to MG.

**FIG 4:**
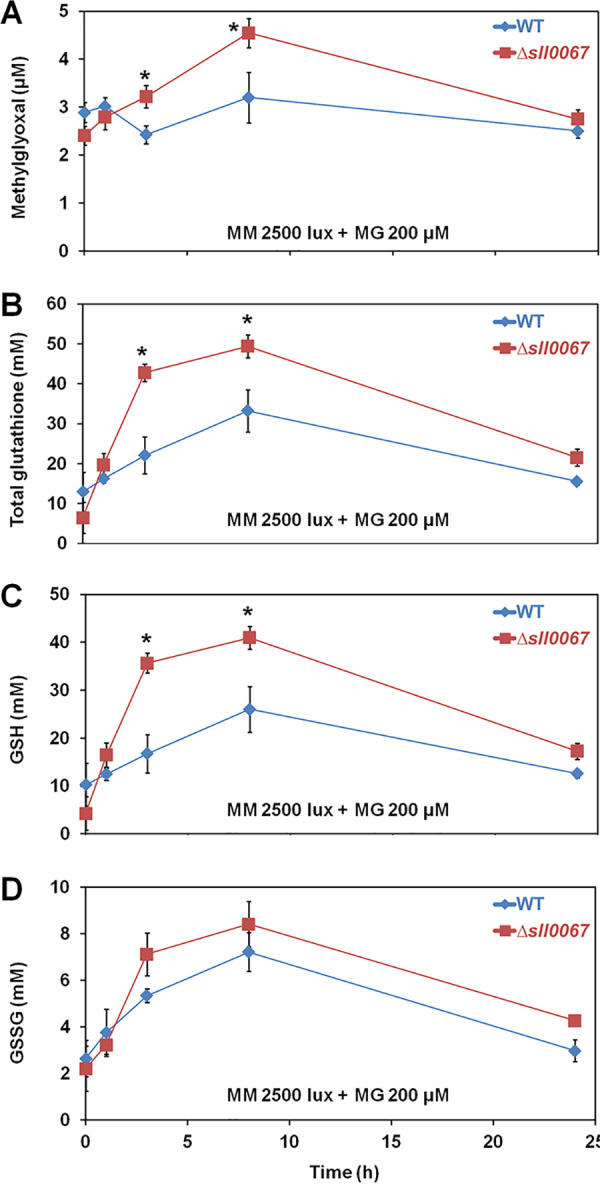
The MG-sensitive Δ*sll0067* mutant exposed to exogenous MG transiently accumulates both methylglyoxal and reduced glutathione. (A) Kinetic accumulation of intracellular MG in WT and Δ*sll0067* mutant challenged by MG. (B to D) Kinetic analysis of the influence of exogenous MG on the abundance of total glutathione and its reduced (GSH) and oxidized (GSSG) forms in WT and Δ*sll0067* cells. The data are expressed as means ± the SD of three experiments. *, Significant difference between WT and Δ*sll0067* cells (*t* test, *P* < 0.05).

Since the main MG detoxification pathway is catalyzed by the GSH-requiring glyoxalase pathway (20), we measured the kinetics of the MG-triggered accumulation of MG and the possible changes in GSH abundance in the WT and Δ*sll0067* strains (Fig. 4). In response to MG, the Δ*sll0067* mutant transiently accumulated more MG and GSH than did the WT strain. These data indicate that Δ*sll0067* cells are sensitive to MG because they have a reduced capability of using GSH to eliminate MG. By extension, these data suggest that Sll0067 normally operates in a GSH-dependent MG removal process such as the glyoxalase system.

### Sll0067 catalyzes the conjugation of GSH with MG.

**FIG 5:**
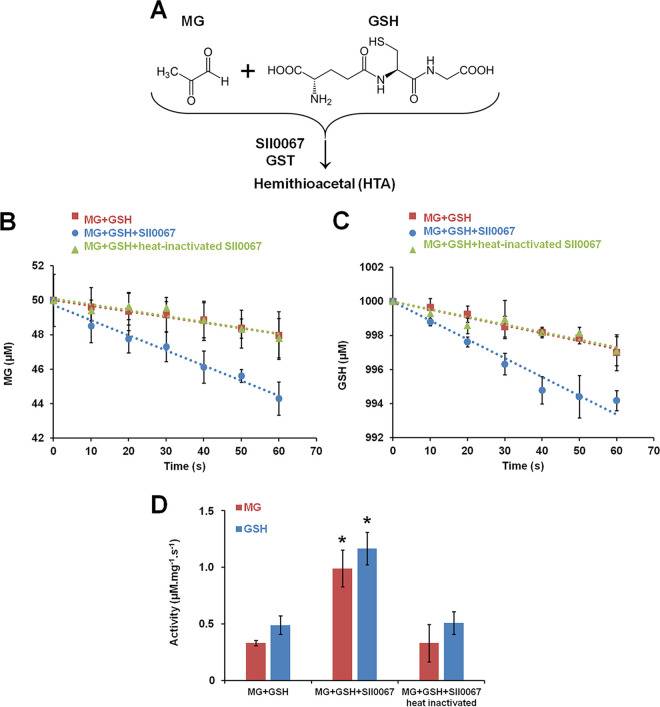
Sll0067 promotes the disappearance of free MG and GSH. (A) Schematic representation of the conjugation of GSH with MG to produce hemithioacetal, a reaction always regarded as spontaneous. (B and C) Influence of Sll0067, before or after heat inactivation, on the kinetics of the disappearance of MG and GSH measured at 30°C in phosphate buffer containing 50 μM MG, 1,000 μM GSH, and 2 μM Sll0067 GST. (D) Decrease in GSH and MG measured in the absence or presence of Sll0067 before or after heat inactivation. Data, expressed as μM mg^−1^ of protein s^−1^, represent means ± the SD of three experiments. *, Significant difference between the decreases of GSH and MG observed in the presence or absence of Sll0067 activity (*t* test, *P* < 0.05).

The above-mentioned *in vivo* findings that Sll0067 operates in resistance to MG and the removal of MG and GSH (Fig. 2 and 4) prompted us to analyze *in vitro* the influence of Sll0067 on MG and GSH (Fig. 5A). A His-tagged recombinant Sll0067 protein was purified as a homodimer product of about 43 kDa (Fig. S3) and found to have a good GSH transferase activity (Table S3) toward the classical GST substrates phenetyl isothiocyanate (PITC), benzyl isothiocyanate (BITC), and 1-chloro-2,4-dinitrobenzene (CDNB) with measured catalytic efficiencies (*k*_cat_/*K_m_*) of 6.7 × 10^5^ M^−1^ s^−1^, 5.7 × 10^5^ M^−1^ s^−1^, and 112.5 M^−1^ s^−1^, respectively. The lower catalytic efficiency observed for CDNB is due to the lower Sll0067 affinity observed for this substrate (3,800.0 μM) compared to the values observed for BITC (82.0 μM) and PITC (31.4 μM). The comparable kinetic parameters obtained for BITC and PITC indicate that the modulation in the aromatic group (benzyl versus phenetyl) does not affect substrate recognition (Table S3).

10.1128/mBio.00882-20.3 TABLE S3 Kinetic parameters of Sll0067 GST. Enzymatic activities were measured as described in the Materials and Methods section, using various concentrations of the following substrates: CDNB (1-chloro-2,4-dinitrobenzene), BITC (benzyl isothiocyanate), and PITC (phenetyl isothiocyanate). Results are presented as means ± the standard deviations of three independent measurements. Download Table S3, DOCX file, 0.01 MB. Copyright © 2020 Kammerscheit et al. 2020 Kammerscheit et al. This content is distributed under the terms of the Creative Commons Attribution 4.0 International license.

10.1128/mBio.00882-20.6 FIG S3 Purification and analysis of the recombinant Sll0067 GST produced in E. coli. (A) Coomassie blue-stained sodium dodecyl sulfate-polyacrylamide gel electrophoresis (SDS-PAGE) analysis of noninduced (NI), total (T), soluble (S), and insoluble (I) protein fractions from E. coli Rosetta2(DE3)/pLysS propagating the Sll0067-producing plasmid grown in the absence or presence of 0.1 mM IPTG. Fractions collected during immobilized metal affinity chromatography (IMAC) and size-exclusion chromatography (SEC) were also analyzed (W1 and W2, washing steps; E, elution). MM, molecular mass marker. (B) Purified Sll0067 protein (300 μg in 300 μl of lysis buffer) that was analyzed by SEC-MALS using an analytical Superdex200 10/300 column connected to a multiangle light scattering (MALS) detector (miniDAWN TREOS; Wyatt Technology) and a refractometer (T-rEX; Wyatt Technology). Data were processed using Astra 7 software (Wyatt Technology). Download FIG S3, TIF file, 0.3 MB. Copyright © 2020 Kammerscheit et al. 2020 Kammerscheit et al. This content is distributed under the terms of the Creative Commons Attribution 4.0 International license.

The capacity of Sll0067 to catalyze the conjugation of GSH with MG was then assayed by measuring the levels of free (unconjugated) GSH and MG as a function of incubation times in the presence or absence of Sll0067. Sll0067 appeared to accelerate the disappearance of free MG and GSH (the conjugation of MG with GSH) over the spontaneous (nonenzymatic) levels observed in the absence of Sll0067 or after heat inactivation of Sll0067 (Fig. 5B to D). This finding is important because the conjugation of GSH with MG, the first step of MG detoxification by the glyoxalase system, is always presented as being spontaneous (nonenzymatic) in both prokaryotes (1) and eukaryotes (2, 3).

**FIG 6:**
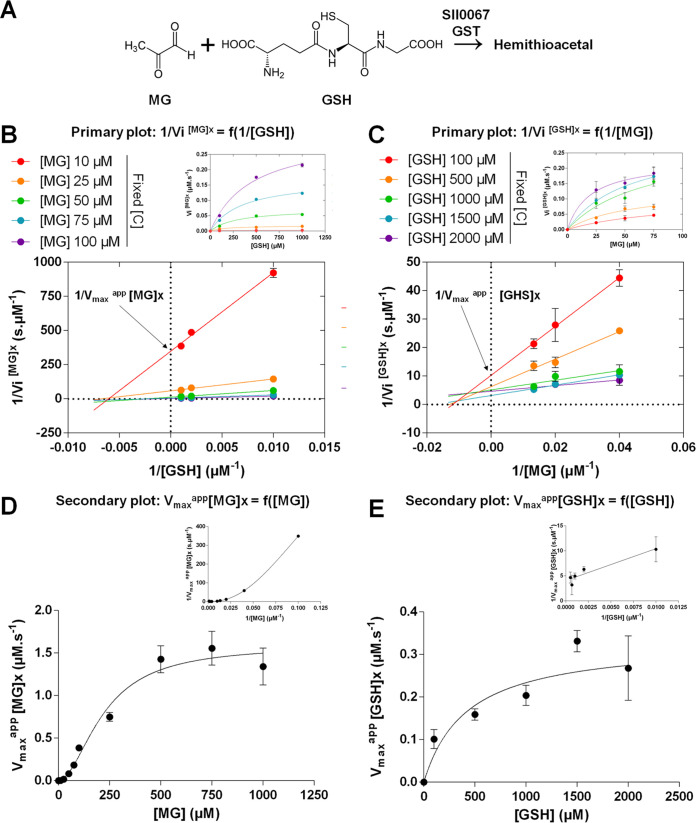
Sll0067 catalyzes the conjugation of MG with GSH by a steady-state sequential mechanism. (A) Schematic representation of the Sll0067-accelerated conjugation of GSH with MG. (B) Initial velocity (primary) plot of Sll0067 reaction with GSH (variable concentrations) and MG (fixed concentrations) with double reciprocal plots of 1/*V*_i_ [MG]*x* versus 1/[GSH], and the corresponding *V*_i_ [MG]*x* = f([GSH]) shown in the upper right corner. (C) Initial velocity plot of Sll0067 activity with GSH (fixed concentrations) and MG (variable concentrations) with double-reciprocal plots of 1/*V*_i_ [GSH]*x* versus 1/[MG], and the corresponding *V*_i_ [GSH]*x* = f([MG]) shown in the upper right corner. (D) Initial velocity (secondary plot) of Sll0067 activity as a function of MG concentration. (E) Initial velocity (secondary plot) of Sll0067 activity as a function of GSH concentration. The data are presented as means ± the SD of three experiments.

The Sll0067-driven disappearance of free GSH and MG was further studied (Fig. 6) using classical two-substrate steady-state kinetic analysis (21). Hence, we measured the initial velocity of MG transformation (conjugation with GSH) at fixed MG levels for various concentrations of GSH and vice versa, yielding the primary plots 1/*V*_i_^[MG]^*^x^* = f(1/[GSH]) and 1/*V*_i_^[GSH]^*^x^* = f(1/[MG]) shown in Fig. 6B and C. The Lineweaver-Burk plots obtained using MG as the fixed substrate and GSH as the variable substrate intersected the *x* axis (Fig. 6B), suggesting that the interaction of Sll0067 with GSH does not modify its affinity for MG. The Lineweaver-Burk plots obtained when MG and GSH were used, respectively, as variable and fixed substrates, intersected above the *x* axis (Fig. 6C), suggesting that the interaction of Sll0067 with MG increases its affinity for GSH.

Then, the secondary plots were constructed from the *y* axis intercept known as 1/*V*_max_^app^[MG] values of each curve (i.e., each fixed MG concentrations) from the primary double-reciprocal pattern and *vice versa* for GSH (Fig. 6B and E). The *V*_max_^app^[MG]*x* = f([MG]) pattern revealed the allosteric behavior of Sll0067 toward MG (Fig. 6D) and suggests a positive K-type cooperativity. The corresponding Hill number n_h_ (1.873 ± 0.261; Table 1) is consistent with the dimeric nature of Sll0067 (22; see also Fig. S3 in the supplemental material), and it validates the cooperative fixation of MG onto Sll0067 (n_h_ > 1). The *V*_max_^app^[GSH]*x* = f([GSH]) pattern revealed the Michaelian behavior of Sll0067 toward GSH (Fig. 6E). These secondary plots were also used to calculate the Sll0067 apparent *K_m_* and *k*_cat_ for GSH, and Hill number n_h_ and *K*_0.5_ for MG (Table 1). The reasonably good catalytic efficiency of Sll0067 for GSH (355.6 M^−1^ s^−1^) suggests that the *K_m_* value for GSH (421.7 ± 70.5 μM; Table 1) is consistent with the high intracellular content in GSH (5 to 20 mM [12, 13]). Furthermore, the kinetic data represented in Fig. 6D suggests that the (allosteric) fixation of MG on Sll0067 increases its affinity for GSH.

**TABLE 1:** Kinetic parameters of Sll0067 activity on both MG and GSH substrates

Parameter	Mean ± SD^*a*^	
	MG	GSH
*k*_cat_ (s^−1^)	NA	0.15 ± 0.02
*K_m_* (μM)	NA	421.70 ± 70.50
*k*_cat_/*K_m_* (M^−1^ s^−1^)	NA	355.60 ± 0.12
*K*_0.5_ (μM)	221.7 ± 26.3	NA
n_h_	1.873 ± 0.261	NA

a Enzymatic activities were measured as described in Materials and Methods using various concentrations of the following substrates MG and GSH. The results are presented as the means of three independent measurements. NA, not applicable.

The relatively high *K*_0.5_ value for MG (221.7 ± 26.3 μM; Table 1) indicates that the positive (K^+^) cooperative fixation of one MG molecule on the first Sll0067 monomer stimulates the fixation of a second MG molecule on the second Sll0067 monomer and the subsequent catalysis. Furthermore, Sll0067 appeared to be more active on MG (catalytic activity 355.6 M^−1^ s^−1^; Table 1) than on CDNB (112.5 M^−1^ s^−1^; Table S3). The presently reported two-substrate kinetic analysis of Sll0067 (Fig. 6B to E) is consistent with the steady-state sequential kinetic mechanism that has been observed for other types of GSTs acting on other substrates (23).

### Sll0067 activity is stimulated by *S*-d-lactoylglutathione, the intermediate product in MG detoxification.

**FIG 7:**
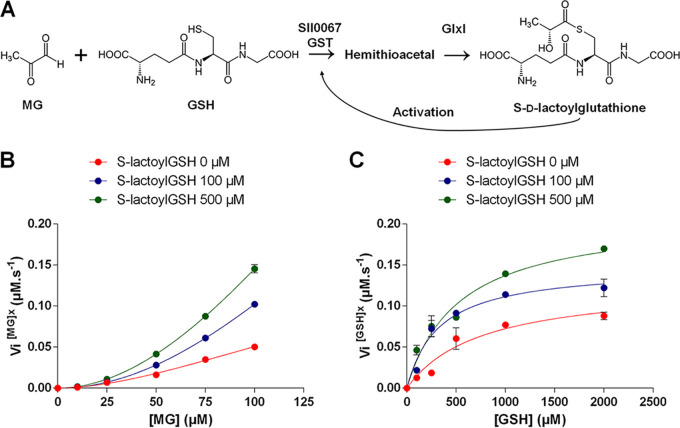
The Sll0067-driven conjugation of MG with GSH is stimulated by the *S*-d-lactoylglutathione intermediate in MG detoxification. (A) Schematic representation of the conjugation of GSH with MG to initiate the glyoxalase-dependent detoxification of MG. (B) Initial velocity graph of Sll0067 activity in the presence of a fixed GSH concentration and the indicated concentrations of MG and *S*-d-lactoylglutathione (S-lactoylGSH). (C) Initial velocity graph of Sll0067 activity with a fixed MG concentration and the indicated concentrations of GSH and S-lactoylGSH. The data are presented as means ± the SD of three experiments.

Our evidence that Sll0067 catalyzes the conjugation of GSH with MG (Fig. 7A), likely yielding the hemithioacetal (HTA) subsequently transformed by GlxI into *S*-d-lactoylglutathione (S-lactoylGSH) (6), together with the crystallization of a Phi-class GST (similar to Sll0067) in the presence of S-lactoylGSH (24), prompted us to test the influence of S-lactoylGSH on the Sll0067-driven conjugation of GSH with MG. All measured initial velocities of Sll0067 activity were increased by S-lactoylGSH (Fig. 7B and C). These data indicate that S-lactoylGSH stimulates the Sll0067-catalyzedconjugationof GSH with MG to facilitate MG detoxification by the glyoxalase pathway.

## DISCUSSION

Glutathione transferases (GSTs) are widespread enzymes known to use glutathione (GSH) for the detoxification of ROS, metabolite by-products, xenobiotics, and/or heavy metals. Attesting to the importance of GSTs, higher organisms possess many GSTs (20, 25), making it difficult to analyze their selectivity or redundancy.

The analysis of GSTs is easier in cyanobacteria, the environmentally crucial prokaryotes (8) regarded as the originators of photosynthesis (10) and GSH-dependent enzymes (11), because they possess few GSTs. For example, the model cyanobacterium *Synechocystis* sp. strain PCC 6803 has only six GSTs (Sll0067, Sll1147, Sll1545, Sll1902, Slr0236, and Slr0605). Furthermore, cyanobacteria have a great potential for an ecological production of industrially interesting chemicals that is often hampered by our limited knowledge of cyanobacterial responses to different stresses (26).

In this study, we analyzed the role of Sll0067 in *Synechocystis* sp. strain PCC 6803. Therefore, a Δ*sll0067*::Km^r^ (Δ*sll0067*) deletion mutant was constructed and appeared to grow as fit as the WT strain in standard (photoautotrophic) conditions. Furthermore, we found that Sll0067 is not involved in the tolerance to either photo-oxidative stress, unlike Sll1545 and Slr0236 (12), or to heat, cold, or lipid peroxidation, unlike Sll1147 (13). Very interestingly, the Δ*sll0067* deletion mutant appeared to be hypersensitive to exogenous MG (Fig. 2), a toxic metabolite by-product (it causes diabetes in humans) (1, 3), unlike all other GST deletion mutants (Δ*sll1147*, Δ*sll1545*, Δ*sll1902*, Δ*slr0236*, and Δ*slr0605*).

The MG-sensitive Δ*sll0067* mutant exposed to MG (or glucose) accumulated MG (Fig. 3), indicating that Sll0067 plays a significant role in MG removal. These findings are welcome because MG has been overlooked in photosynthetic organisms, even though they inevitably produce MG not only by the catabolism of sugars, amino acids, and lipids, like heterotrophic organisms (from E. coli to humans) but also by their active photosynthetic assimilation of CO_2_ (2). This issue is even more acute in cyanobacteria that perform photosynthesis (fixation of CO_2_ and gluconeogenesis) and respiration (glucose catabolism) in the same cell compartment (6).

The Δ*sll0067* mutant challenged by MG accumulated not only MG but also GSH (Fig. 4), indicating that Sll0067 operates in a MG elimination process that requires GSH, similarly to the GSH-dependent detoxification of MG catalyzed by the glyoxalase system. This interpretation was confirmed by *in vitro* tests showing that Sll0067 catalyzes the conjugation of GSH with MG (Fig. 5). This finding is interesting because in most organisms MG is mainly detoxified by the GSH-dependent glyoxalase pathway that begins with the conjugation of MG and GSH, a reaction always presented as spontaneous (nonenzymatic). The resulting hemithioacetal metabolite is then isomerized by GlxI and hydrolyzed by GlxII to release d-lactate and GSH (3, 4).

Our data also indicate that the cooperative fixation of one MG molecule on the first subunit of the Sll0067 dimeric enzyme stimulates the fixation of a second MG molecule on the second Sll0067 monomer (Fig. 6), thereby increasing Sll0067 activity. We also found that the fixation of MG on Sll0067 enhances its affinity for GSH (Fig. 6) and that Sll0067 is also activated by S-d-lactoylGSH (Fig. 7), the intermediate product in MG detoxification. All of these findings indicate that MG enhances the Sll0067-driven conjugation of GSH and MG to promote MG detoxification by the glyoxalase pathway. They will undoubtedly stimulate research on MG signaling and detoxification in animals and humans (with possible implications on identification of biomarkers and drugs), plants (with possible influence on agriculture), and cyanobacteria (with probable implications on the production of industrially interesting carbon-based chemicals). Last, but not least, our evidence that Sll0067 acts in the detoxification of MG, involved in diabetes in humans, is consistent with the existence of a correlation between the occurrence of diabetes and the (poor) activity of a human GST homologous to Sll0067 (27).

## MATERIALS AND METHODS

### Bacterial strains, growth, and stress assays.

Escherichia coli strains used for gene manipulations were grown at 37°C in LB culture medium containing the selective antibiotics: ampicillin (Amp) ay 100 μg ml^−1^ and kanamycin (Km) at 50 μg ml^−1^ (Top10 strain; Invitrogen) or Km at 50 μg ml^−1^ and chloramphenicol at 34 μg ml^−1^ [Rosetta2(DE3)/pLysS strain; Novagen].

*Synechocystis* sp. strain PCC 6803 was routinely grown at 30°C in liquid mineral medium (MM) under white light (2,500 lx; 31.25 μE m^−2^ s^−1^) as described previously (13). The deletion mutants were grown in the presence of the selective antibiotic (Km, 50 μg ml^−1^). For growth analysis of the MG effect, mid-exponential-phase cultures (optical density at 580 nm [OD_580_] = 0.3 to 0.8) adjusted to an OD_580_ of 0.02 (5 × 10^5^ cells ml^−1^) were incubated in MG-containing liquid MM prior to measuring the OD_580_ or photographing the culture flasks. For survival analyses, 10-ml portions of mid-exponential-phase cultures (adjusted to OD_580_ 0.1) were challenged with MG, serially diluted in MM, spread on MM solidified with 1% agar (Difco), and incubated (for 5 to 7 days) under standard conditions before counting the colonies generated by viable cells.

### Targeted deletion of the *sll0067* gene.

The Δ*sll0067*::Km^r^ deletion cassette was constructed by replacing the full *sll0067* coding sequence (CS) by a transcription-terminator-less kanamycin resistance gene (Km^r^) for selection, while preserving the *sll0067*CS flanking DNA regions for homologous recombination mediating targeted gene replacement in *Synechocystis* sp. strain PCC 6803 (15). These DNA regions (about 300 bp) amplified by PCR, using specific primers (Table S2), were joined by PCR-driven overlap extension on both sides of a SmaI restriction site and cloned in pGEMT (Table S1). The resulting plasmid (Table S1) was opened at its unique SmaI site where we cloned the Km^r^ gene (a HincII fragment of pUC4K) in the same orientation as the sll0067 CS it replaced. The resulting deletion cassette Δ*sll0067*::Km^r^ was verified by PCR and DNA sequencing (Mix2Seq kit; Eurofins Genomics) before and after transformation (15) to *Synechocystis* sp. strain PCC 6803.

### Glutathione assay.

This assay was performed strictly as previously described (12, 13). Cells were rapidly collected by filtration, resuspended in an acidic phosphate buffer and disrupted by freezing-thawing cycles. Cell extracts were purified by centrifugation through an Amicon filter and stored at −80°C. Cell extracts treated with 2-vinylpyridine and triethanolamine to block reduced glutathione (GSSG assays) and untreated samples (total glutathione [GSH+GSSG] assays) were incubated with yeast glutathione reductase, NADPH, and DTNB [5,5′-dithiobis-(2-nitrobenzoic acid)] prior to measuring the absorption at 412 nm of TNB (5′-thio-2-nitrobenzoic acid). Standard curves prepared with various concentrations of GSH or GSSG were used to calculate the cell content in GSSG, total glutathione, and GSH (total glutathione minus GSSG) using the *Synechocystis* sp. strain PCC 6803 cell volume of 1.2 × 10^−11^ ml (28).

### Methylglyoxal assay.

Next, 100 ml of exponentially growing cultures were diluted to an OD_580_ of 0.6 and incubated under white light (2,500 lx) in the presence of 200 μM MG (Sigma-Aldrich). Cells were rapidly collected by filtration on a 0.45-μm cellulose membrane (Millipore) under light, resuspended in 1 ml of ultrapure water (UPW), disrupted by three freeze-thaw cycles in liquid nitrogen and a hot water bath, and strong mixing (Vibrax VXR; Ika) for 10 min at 4°C prior to centrifugation (14,000 rpm, 4°C, 5 min) to eliminate unbroken cells and membranes. Cell extracts were purified by a 20-min centrifugation (14,000 rpm, 4°C) through a filter (Amicon Ultra; 0.5 ml 30K; Millipore) to eliminate proteins larger than 30 kDa and stored at −80°C. MG assays (16, 17) were conducted in reaction mixtures adjusted to pH 10.0 with 1.0 M NH_4_Cl/NH_3_ (pH 10.0) buffer (Merck) and a 20:1 ratio of DDP (5,6-diamino-2,4-dihydroxypyrimidine sulfate dihydrate; Combi-Blocks) over MG. Then, 1-ml samples were mixed with 0.5 ml of alkaline ammonium buffer (see above) and 2 ml of derivatizing agent (1 mM DDP), heated for 120 min at 60°C, cooled, and loaded onto a microplate. The fluorescence of the MG-DDP derivative (λ_exc_ = 362 nm and λ_em_ = 445 nm) was measured with a microplate spectrofluorimeter (ClarioStar; BMG Labtech). Standards prepared with various MG concentrations (1 to 7.5 μM in UPW) were used to calculate the intracellular MG content.

### Production and purification of the His-tagged Sll0067 recombinant protein.

The *sll0067*CS was cloned (NEBuilder HiFi DNA assembly master mix; New England BioLabs) as an NdeI-NcoI DNA segment into the pET-26b plasmid (Table S1) linearized with NdeI and NcoI. The resulting plasmid pET-26b(+)-*sll0067*, verified by DNA sequencing, was transformed to E. coli Rosetta2(DE3)/pLysS cells (Table S1). The production of Sll0067 was induced in cells grown at 37°C in LB+Km+Cm at an OD_600_ of 0.7 to 0.8 with 0.1 mM IPTG (isopropyl-β-d-1-thiogalactopyranoside) for 4 h. Cells were harvested by centrifugation, resuspended in lysis buffer (30 mM Tris-HCl [pH 8.0], 200 mM NaCl), and stored at −20°C prior to sonication and centrifugation (35,000 × *g*, 25 min, 4°C) to collect the supernatant. The (C-terminal) His-tagged Sll0067 protein was purified by affinity chromatography on a nickel nitrilotriacetate (Ni-NTA) agarose resin (Qiagen), followed by size exclusion chromatography on a preparative Superdex 200 16/600 column (equilibrated in 30 mM Tris-HCl [pH 8.0], 200 mM NaCl) connected to an ÄKTA purifier (GE Healthcare). The protein concentration was determined by measuring the absorbance at 280 nm and by using a specific extinction coefficient of 28,420 M^−1^ cm^−1^.

To determine the oligomerization state of Sll0067, 300 μg of purified protein in 300 μl was injected at a flow rate of 0.5 ml/min on an analytical Superdex 200 10/300 column (equilibrated in 30 mM Tris-HCl [pH 8.0]–200 mM NaCl connected to an ÄKTA purifier equipped with a multiangle light scattering detector (miniDAWN TREOS; Wyatt Technology) and a refractometer (T-rEX; Wyatt Technology). Data were processed using Astra 7 software (Wyatt Technology).

### Enzymatic activity of Sll0067 GST.

GSH-conjugations on benzyl isothiocyanate (BITC), phenetyl isothiocyanate (PITC), or 1-chloro-2,4-dinitrobenzene (CDNB) were assayed by monitoring the absorbance at 274 nm (BITC or PITC) or 340 nm (CDNB) as described previously (29). Reactions were carried out at 25°C in 500 μl of 100 mM phosphate buffer (pH 6.5; BITC or PITC) or 30 mM Tris-HCl (pH 8.0)–1 mM EDTA (CDNB). Various concentrations of BITC, PITC, and CDNB were tested at a fixed 1 mM GSH concentration. Reactions were started by the addition of a 2.21 μM Sll0067 concentration, yielding a linear response range. Measured velocities were corrected by subtracting the rate of the spontaneous reaction (absence of Sll0067). Three independent experiments were performed at each substrate concentration. The kinetic parameters (*k*_cat_ and apparent *K_m_*) were obtained by fitting the data to the nonlinear regression Michaelis-Menten model in Prism 8 software (GraphPad). The *k*_cat_ values are expressed as μmol of substrate oxidized per s per μmol of enzyme, using specific molar absorption coefficients of 6,220 M^−1^ cm^−1^ at 340 nm for NADPH, 8,890 M^−1^ cm^−1^ at 274 nm for PITC, 9,250 M^−1^ cm^−1^ at 274 nm for BITC, and 9,600 M^−1^ cm^−1^ at 340 nm for CDNB.

### Enzymatic assays of GSH and MG depletions.

The disappearance of MG (50 μM) and GSH (1000 μM) catalyzed by Sll0067 (2.21 μM) was analyzed at 30°C in 100 mM KH_2_PO_4_/K_2_HPO_4_–1 mM EDTA (pH 7.5). Three independent experiments were performed for each time of reaction, which were started by the addition of both MG and Sll0067 and stopped by the addition of ice-cold 0.5 ml of 1.0 M NH_4_Cl/NH_3_ (pH 10.0; Merck). Samples were stored, prior to measuring the remaining MG as described above. GSH-consuming reactions were stopped by the addition of 1 ml of 100 mM KH_2_PO_4_/K_2_HPO_4_, 1 mM EDTA, and 1.2% (wt/vol) 5-sulfosalicylic acid. Then, 5 μl of the reaction mixture was loaded onto a microplate, and the remaining concentration of GSH was measured as described above. The initial velocity of Sll0067 reaction was expressed as μM s^−1^ mg^−1^ of enzyme.

### Two-substrate kinetic analysis of Sll0067 GST.

Steady-state kinetic of Sll0067-driven consumption of GSH and MG were performed at 30°C in 1 ml of 100 mM KH_2_PO_4_/K_2_HPO_4_ and 1 mM EDTA (pH 7.5). Initial velocities were determined by assaying variable MG concentrations at fixed GSH concentrations and vice versa. The reactions were started by adding both MG and Sll0067 (2.21 μM), and three independent experiments were performed at each substrate concentration. The measured velocities were corrected by subtracting the rate of the spontaneous reactions (absence of Sll0067). The kinetic parameters (*k*_cat_ and apparent *K_m_*) and allosteric parameters (Hill number n_h_ and *K*_0.5_) were obtained by fitting the data to the nonlinear regression Michaelis-Menten model and to the allosteric sigmoidal model in GraphPad Prism 6 software, respectively.
