# Supplementary material for: Methylglyoxal Detoxification Revisited: Role of Glutathione Transferase in Model Cyanobacterium Synechocystis sp. Strain PCC 6803
Source: mBio. 2020 Aug 4;11(4):e00882-20. doi: 10.1128/mBio.00882-20 (PMC7407080; doi:10.1128/mBio.00882-20)
Supplement: TABLE S1 [file mBio.00882-20-st001.docx]

**Table S1. Characteristics of the genes and plasmids used in this study.**

|  | | |
| --- | --- | --- |
| **Plasmid** | **Relevant features** | **Reference** |
| **pGEMT** | Amp^r^ AT overhang cloning vector | Promega |
| **pUC4K** | Source of the Km^r^ marker gene | Pharmacia |
| **pFC1** | Source of the Sm^r^/Sp^r^ marker gene | (1) |
| **pET-26b(+)** | Km^r^ *E. coli* expression vector |  |
| **pGEMT-AB*sll0067*** | pGEMT with the *sll0067*locus, where most of the *sll0067* coding sequence (from the 3^rd^ bp downstream of the ATG codon down to the stop codon) was replaced by a *Sma*I restriction site | This study |
| **pGEMT-Δ*sll0067*::Km^r^** | pGEMT-AB*sll0067* with the Km^r^ marker inserted in the unique *Sma*I site | This study |
| ***Nde*I-*sll0067*-*Xho*I (synthetic)** | Synthetic DNA fragment (Twist Bioscience) containing the 6xHis-tagged*sll0067*coding sequence flanked by the *Nde*I and *Xho*I restriction sites for cloning inpET-26b(+) | This study |
| **pET-26b(+)-*sll0067*** | pET-26b(+) that produces the 6xHis-tagged Sll0067 protein | This study |
| 1. (30)**.** A conditional expression vector for the cyanobacteria *Synechocystis* sp. strains PCC6803 and PCC6714 or *Synechococcus* sp. strains PCC7942 and PCC6301. *Curr. Microbiol.* 28, 145–148. | | |
