# Supplementary material for: Methylglyoxal Detoxification Revisited: Role of Glutathione Transferase in Model Cyanobacterium Synechocystis sp. Strain PCC 6803
Source: mBio. 2020 Aug 4;11(4):e00882-20. doi: 10.1128/mBio.00882-20 (PMC7407080; doi:10.1128/mBio.00882-20)
Supplement: TABLE S2 [file mBio.00882-20-st002.docx]

|  | | |
| --- | --- | --- |
| **Table S2. List and characteristics of the PCR primers used in this study.** | | |
|  | | |
| **Amplification of the Km^r^ cassette prior to its cloning as a *Hinc*II fragment** | | |
| KmHincII-FW | GGCGCTGAGGTCGACCTCGTGAAGAAG |  |
| KmHincII-RV | ACCTGCAGGGGGTCGACGGAAAGCCAC |  |
| **Construction of theΔ*sll0067*::Km^r^ deletion cassette** | | |
| L0067-FWa | GGAGCGCATCGAACCAAA | Amplification of the chromosome region downstream of the *sll0067* coding sequence (CS) |
| L0067-RVa | **TTGTTCCCGGGTACTA**CATGATTTTTTCTCCGATT | Amplification of the chromosome region downstream of the *sll0067* CS and simultaneous introduction of a *Sma*I sitefor cloning the Km^r^ marker as a *Hinc*II fragment |
| L0067-FWb | **TAGTACCCGGGAACAA**CCAGTGACCAGATCAACCT | Amplification of the chromosome region upstream of the *sll0067*CS and simultaneous introduction of a *Sma*I site for cloning the Km^r^ marker as a*Hinc*II fragment |
| L0067-RVb | TTCTTGACCGTGGTGATTGTTC | Amplification of the chromosome region upstream of the *sll0067*CS |
| **Amplification of the Km^r^ marker to verify the segregation between WT and Δ*sll0067*::Km^r^ chromosomes in the Δ*sll0067* mutant** | | |
| KmFW **(3)** | GGTGTTATGAGCCATATTCAACGGG |  |
| KmRV **(4)** | GGGAAGATGCGTGATCTGATCCTTC |  |
| **Amplification of the *sll0067* gene to verify the segregation between WT and Δ*sll0067*::Km^r^ chromosomes in the Δ*sll0067* mutant** | | |
| SLL0067-FW **(1)** | CGTTAACCTCAAGGAAGGGG |  |
| SLL0067-RV **(2)** | CCACTGGTTAACTAGAGCTC |  |
| **Verification of the segregation between WT and Δ*mgs*::Km^r^ chromosome in the Δ*mgs*::Km^r^ mutant** | | |
| SLL0036-FW**(5)** |  |  |
| SLL0036-RV **(6)** |  |  |
| Km2-RV **(7)** |  |  |
| **Amplification of the *sll0067* gene cloned inthe pET-26b(+) plasmid** | | |
| T7-FW | TAATACGACTCACTATAGGG |  |
| T7-RV | GCTAGTTATTGCTCAGCGG |  |
| **Abbreviations:** FW: forward; RV: reverse. The numbers indicated in brackets correspond to those used in supplemental figures (Fig.S1 and Fig. S2). | | |
