# Supplementary figures and images for: Methylglyoxal Detoxification Revisited: Role of Glutathione Transferase in Model Cyanobacterium Synechocystis sp. Strain PCC 6803
Source: mBio. 2020 Aug 4;11(4):e00882-20. doi: 10.1128/mBio.00882-20 (PMC7407080; doi:10.1128/mBio.00882-20)

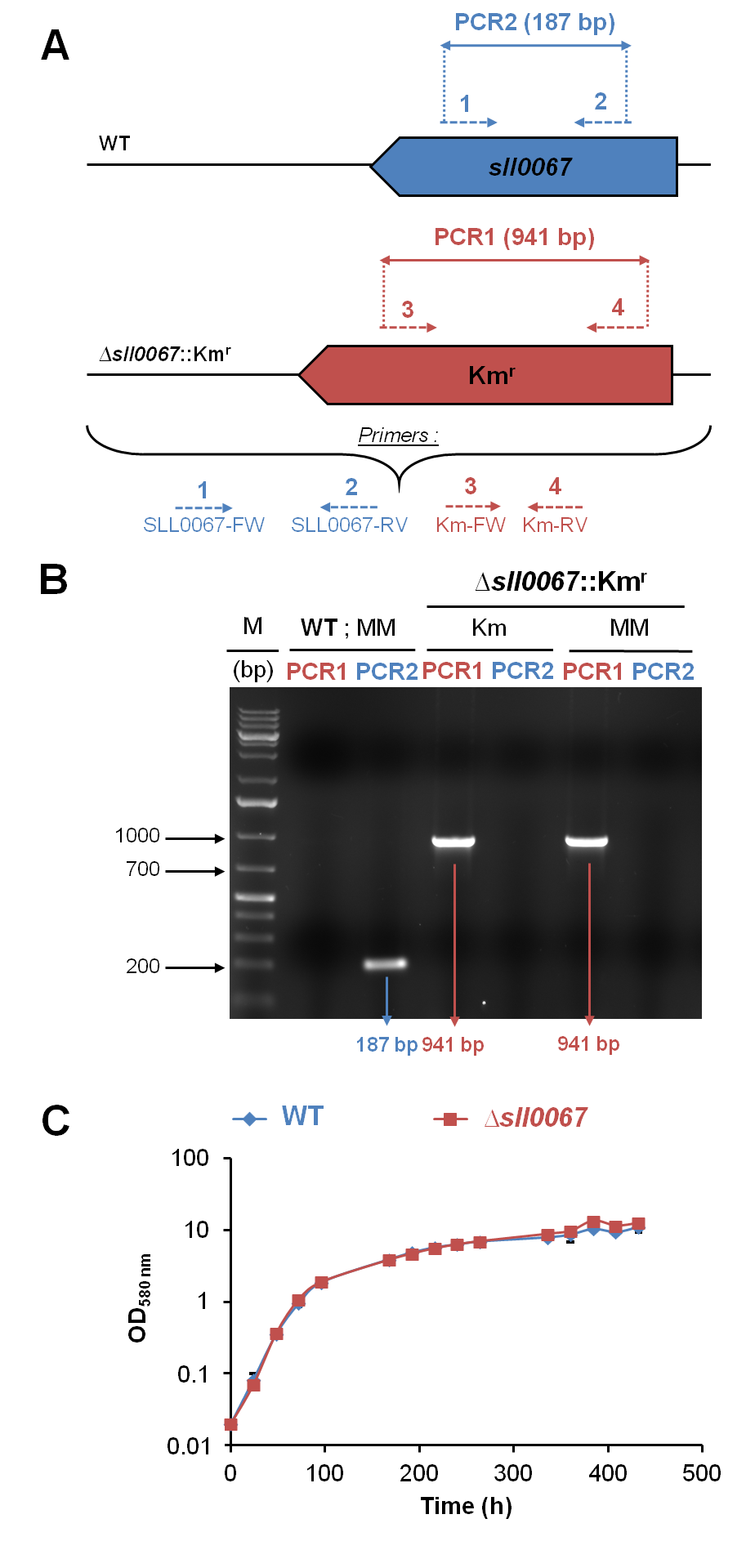

Supplement: FIG S1 [file mBio.00882-20-sf001.tif]

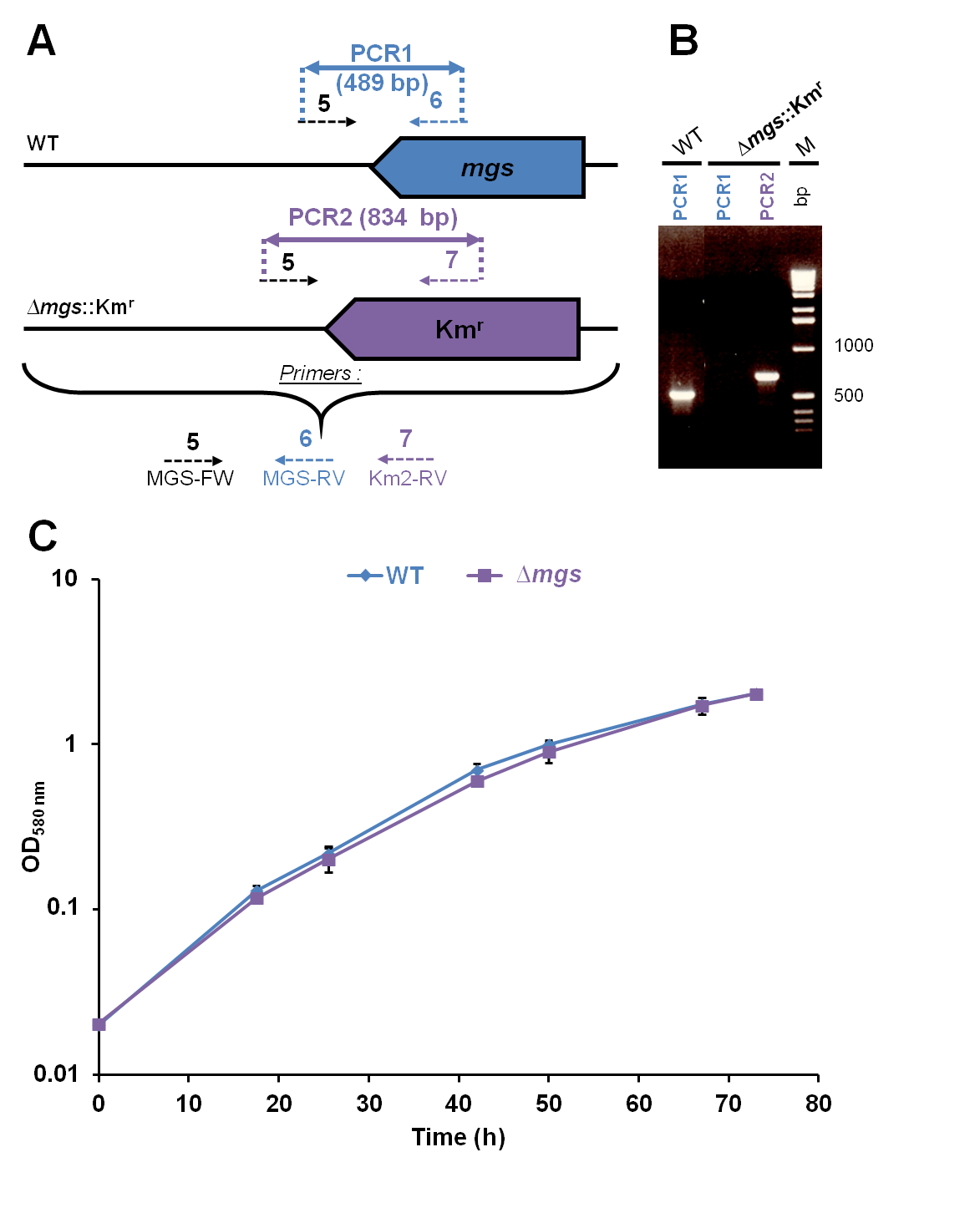

Supplement: FIG S2 [file mBio.00882-20-sf002.tif]

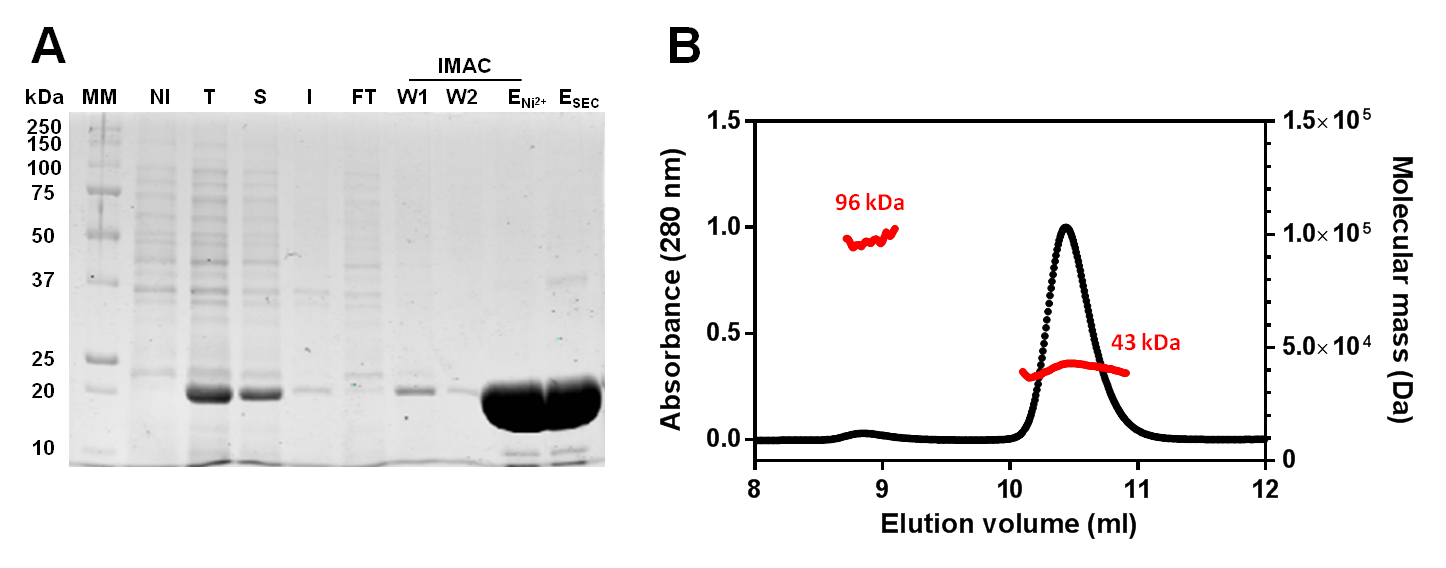

Supplement: FIG S3 [file mBio.00882-20-sf003.tif]
