# Supplementary material for: Methylglyoxal Detoxification Revisited: Role of Glutathione Transferase in Model Cyanobacterium Synechocystis sp. Strain PCC 6803
Source: mBio. 2020 Aug 4;11(4):e00882-20. doi: 10.1128/mBio.00882-20 (PMC7407080; doi:10.1128/mBio.00882-20)
Supplement: TABLE S3 [file mBio.00882-20-st003.docx]

**Table S3. Kinetic parameters of Sll0067 GST.**

|  | **CDNB** | **BITC** | **PITC** |
| --- | --- | --- | --- |
| **k_cat_(s^-1^)** | 0.7 ± 0.2 | 45.0 ± 1.6 | 21.0 ± 0.5 |
| **K_m_ (µM)** | 3800.0 ± 772.2 | 82.0 ± 10.0 | 31.4 ± 3.5 |
| **k_cat_/ K_m_ (M^-1^.s^-1^)** | 112.5 ± 14.2 | 5.7x10^5^ ± 0.2x10^5^ | 6.7x10^5^ ± 0.2x10^5^ |

Enzymatic activities were measured as described in the Materials and methods section, using various concentrations of the following substrates CDNB (1-chloro-2,4-dinitrobenzene), BITC (benzyl isothiocyanate), PITC (phenetyl isothiocyanate). Results are presented as the means ± standard deviation of three independent measurements.
